# Intraaortic counterpulsation in a second-level institution: indications, management and outcome

**DOI:** 10.1186/cc12161

**Published:** 2013-03-19

**Authors:** R Gómez López, P Fernández Ugidos, P Vidal Cortés, M Lorenzo Lage, A Tizón Varela, E Rodríguez Álvarez

**Affiliations:** 1 Complexo Hospitalario Universitario de Ourense, Spain

## Introduction

Counterpulsation is an important support for patients with cardiac diseases. The use of these devices has been limited typically to hospitals with a cardiac surgery service. The aim of this study is to describe the management of patients with an intraaortic balloon pump (IABP) in our second-level institution.

## Methods

An observational study that includes all patients with IABP in our hospital from January 2010 to September 2012, followed to November 2012. Epidemiological and clinical variables as an indication of IABP, therapeutic management and outcome were collected. Because of the small size sample, statistical analysis was limited to descriptive parameters.

## Results

Seventeen patients (76.5% men, age 66.6 ± 11.6 (43 to 85) years, APACHE score 21.5 ± 14.9 (3 to 46) points) were treated with IABP. The reason for ICU admission was STEMI (*n = *6 patients, 35.3%), no-STEMI (*n = *5, 29.4%), acute heart failure (*n = *4, 23.5%) and out-of-hospital cardiac arrest (*n = *2, 11.8%). The indications of IABP were refractory cardiogenic shock (*n = *12, 70.6%), high-risk percutaneous coronary intervention (*n = *2, 11.8%), refractory angina (*n = *1, 5.9%), refractory pulmonary edema (*n = *1, 5.9%) and electrical storm (*n = *1, 5.9%). Five IABPs (27.4%) were inserted in the ICU and the rest in the catheterization laboratory. Six patients (35.3%) suffered a cardiac arrest prior to hemodynamic stabilization. Three patients (17.6%, the electrical storm and the out-of-hospital cardiac arrest) died before coronary catheterization was performed and the other three were treated with mild therapeutic hypothermia. Thirteen patients (76.5%) needed invasive mechanical ventilation. In six patients (35.3%) invasive hemodynamic monitoring was performed (one pulmonary artery catheter, five PICCO). Transthoracic echocardiography was performed in all patients and transesophageal in six (35.3%). Six of the patients (35.3%) were transferred to the reference centre for immediate coronary artery bypass grafting (CABG). No complications were reported during the transfer. During the ICU stay, femoral artery pseudoaneurysm was reported in one of the patients and inguinal hematoma after IABP withdrawal in two (no transfusion required). Four patients (27.4%) died because of refractory cardiogenic shock despite revascularization. The length of mechanical support was 1.67 ± 1.5 days (0 to 6).

## Conclusion

In our hospital the IABP was mainly used in refractory cardiogenic shock because of myocardial ischemia, with an all-cause mortality of 41.2%. Low rates of complications were observed. Transfer of patients with IABP was performed safely.

